# Multivariate estimation of substructure amplitudes for a single-wavelength anomalous diffraction experiment

**DOI:** 10.1107/S2059798323001997

**Published:** 2023-03-28

**Authors:** Navraj S. Pannu, Pavol Skubák

**Affiliations:** a Department of Infectious Diseases, Leiden University Medical Center, Albinusdreef 2, 2333 ZA, Leiden, The Netherlands; Lund University, Sweden

**Keywords:** substructure determination, experimental phasing, multivariate statistics, direct methods, single-wavelength anomalous diffraction, *Afro*

## Abstract

To determine a substructure from single-wavelength anomalous diffraction (SAD) data using Patterson or direct methods, the substructure-factor amplitude (|*F*_a_|) is first estimated. Currently, the absolute value of the Bijvoet difference is widely used as an estimate of |*F*_a_| values for SAD data. Here, an equation is derived from multivariate statistics and tested that takes into account the correlation between the observed positive (*F* ^+^) and negative (*F* ^−^) Friedel pairs and *F*_a_ along with measurement errors in the observed data. The multivariate estimation of |*F*_a_| has been implemented in a new program, *Afro*. Results on over 180 test cases show that *Afro* provides a higher correlation to the final substructure-factor amplitudes (calculated from the refined, final substructures) than the Bijvoet differences and improves the robustness of direct-methods substructure detection.

## Abstract 2

A new equation for the calculation of substructure-factor amplitudes for substructure detection from a single-wavelength anomalous diffraction experiment produces better results compared with the currently used estimates in test cases.

## 1. Introduction

In determining a macromolecular crystal structure solely from its anomalous signal, the first step is to determine the position of the anomalous substructure that is present. The application of direct methods combined with Patterson techniques, as implemented, for example, in the programs *SHELXD* (Schneider & Sheldrick, 2002 ▸) and *HySS* (Grosse-Kunstleve & Adams, 2003 ▸), or the application of phase-retrieval techniques as implemented in *PRASA* (Skubák, 2018 ▸) have proven to be very powerful in detecting anomalous substructures, particularly when the anomalous substructure contains many atoms or the signal is very weak.

In all of these approaches, in order to detect the anomalous substructure an estimate of the substructure-factor amplitude |*F*_a_| is required. The absolute value of the Bijvoet difference (Δ*F* = ||*F* ^+^| − |*F* ^−^||) is typically input to substructure-detection programs as an estimate for |*F*_a_|.

To improve the methods further, here we propose new formulas and a new refinement strategy to calculate |*F*_a_| values. Previously, Terwilliger (1994 ▸) and Burla *et al.* (2002 ▸, 2003 ▸) employed Bayesian and multivariate approaches to obtain the probability distribution of |*F*_a_|. Here, we expand on their work and derive a probability distribution for *P*(|*F*_a_|; |*F* ^+^|, |*F* ^−^|) that takes into account measurement errors in |*F* ^+^| and |*F* ^−^| and does not assume any relationship between the Friedel phases. We report that at least in our practical implementation, better results were obtained by using the approximation of Burla and coworkers, probably due to numerical stability issues of the more general equation. Furthermore, we propose the maximum-likelihood refinement of errors and scale parameters to obtain the optimal values, given the distributions that we have obtained. Finally, we apply the newly implemented |*F*_a_| estimation to over 180 test cases and show the superior performance of these estimates compared with the Δ*F* values when used by the substructure-determination program *PRASA*.

## 2. Methods

To obtain an estimate of the substructure-factor amplitude |*F*_a_| from a SAD experiment, the expected value of |*F*_a_| given the observations |*F* ^+^| and |*F* ^−^| is required. Let *F* ^+^ denote a structure factor with Miller indices *h*, *k*, *l*; (*F* ^−^)* denote the complex conjugate of a structure factor with Miller indices −*h*, −*k*, −*l*; *F*_a_ denote a substructure factor with Miller indices *h*, *k*, *l*; and α^+^ and α^−^ denote the phases of *F* ^+^ and (*F* ^−^)*, which we will refer to as Friedel pair phases. Then, assuming a complex multivariate Gaussian distribution for *P*[*F*_a_, *F* ^+^, (*F* ^−^)*], the following expression can be obtained: 

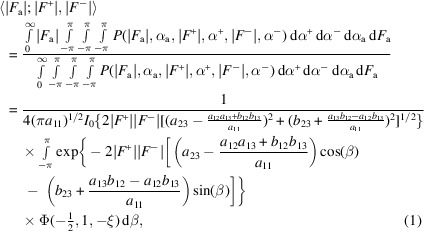

where

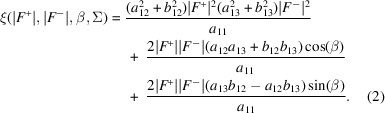




The above expression is derived in Appendix *A* [App appa]; it does not assume α^+^ = α^−^ as was required in earlier publications (Burla *et al.*, 2002 ▸, 2003 ▸), it incorporates the effect of measurement errors in the observed Friedel pair amplitudes and it can be calculated by a single numerical integration. In the above expression, Σ is the (Hermitian) covariance matrix of the complex Gaussian distribution *P*[*F*_a_, *F* ^+^, (*F* ^−^)*], with the elements of its inverse denoted *z*_*jk*_ = *a*_*jk*_ + *ib*_*jk*_, β = α^+^ − α^−^, Φ(*x*, *y*, *z*) is the Kummer confluent hypergeometric function and *I*_0_ is the modified Bessel function of the first kind and of zero order. The covariance matrix Σ was calculated using the expressions derived previously (Pannu *et al.*, 2003 ▸) and the correlation between structure factors. To ensure that the matrix remains positive definite, the inverse of the covariance matrix was calculated from the eigenvalues and eigenvectors calculated from *LAPACK* routines (Anderson *et al.*, 1999 ▸) to remove negative eigenvalues.

We have implemented two equations based on equation (1) in a new program *Afro* for the multivariate estimation of |*F*_a_| values. One equation is equation (1) itself, while the other is a simplified form of equation (1) using the Friedel pair phase equality assumption as suggested by Burla *et al.* (2002 ▸, 2003 ▸): 

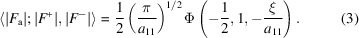

We have found that the simpler equation (3), *i.e*. assuming that the Friedel pair phases are equal, led to better performance in the test cases shown below, which is likely to be due to improved numerical stability. Thus, results from the implementation of this equation are shown below.

The covariance matrix Σ depends on both the number and the (overall) temperature factor of the substructure atoms. As these parameters are usually unknown, a likelihood estimate is obtained by *Afro*. Thus, after initial estimates of the number and the overall temperature factor of the substructure atoms have been input, the parameters are refined using the marginal distribution *P*(|*F* ^+^|, |*F* ^−^|). The refinement of these parameters turned out to have a large radius of convergence, and better results were obtained when refined values were used compared with when unrefined values. We have previously discussed the procedure (Pannu, 2007 ▸) and a similar approach was recently reported by Hatti *et al.* (2021 ▸). After the refinement, the |*F*_a_| values are estimated using equation (1). Local scaling (Blessing, 1997 ▸) has been also implemented in *Afro* which scales |*F* ^+^| to |*F* ^−^| in local spheres.

The multivariate |*F*_a_| calculation using the Friedel pair phase equality assumption as implemented in *Afro* was tested on a sample of 182 SAD data sets as specified in Appendix *B* [App appb] containing a large number of anomalous scatterers (selenium, sulfur, iodine, zinc, gold, copper, platinum, krypton, manganese, iron, cadmium, nickel, calcium and mercury) and a large range of data resolutions from 0.94 to 3.9 Å. For each data set, a complete *Crank*2 (Skubák & Pannu, 2013 ▸) structure-solution run was performed, with *Afro* being used for the calculation of |*F*_a_| and *E* (normalized |*F*_a_|), *PRASA* being used for substructure determination and *REFMAC*5 (Nicholls *et al.*, 2018 ▸), *Parrot* (Cowtan, 2010 ▸), *Buccaneer* (Cowtan, 2008 ▸) and *SHELXE* (Usón & Sheldrick, 2018 ▸) being used in the subsequent combined phasing, density modification and model building. Versions of the programs corresponding to *CCP*4 (Winn *et al.*, 2011 ▸) version 8.0.002 were used, except for *Crank*2, where the more recent version 2.0.325 was used, and a bug fix in *REFMAC*5 implemented by us to prevent the program from crashing for very large data sets.

The input to *Crank*2 consisted of the SAD data set, the protein sequence and a specification of the anomalously scattering atom type with anomalous scattering coefficients. For five data sets, a value of the solvent content corresponding to the correct number of monomers in the asymmetric unit was specified, otherwise the default options were used. An incorrect solvent-content estimate would not affect the |*F*_a_| estimation as it is not used in it; however, since it is an important phase-improvement parameter, it would lead to ‘randomly’ incomplete models for data sets that could otherwise be automatically built, thus making the model-building analysis less relevant.

For each data set we calculated the overall correlation of the estimated *E* values with the ‘final’ substructure *E* values in the following way. The final anomalously scattering substructure (either deposited or, if not available, determined from the anomalous difference maps) was input to *REFMAC*5 using 0 refinement cycles. The calculated amplitudes from *REFMAC*5 were then input to *ECALC* from *CCP*4 (Ian Tickle, unpublished work), providing the final substructure *E* values. The correlation between the estimated and final *E* values was calculated using the *SFTOOLS* utility from *CCP*4 (Bart Hazes, unpublished work), which divided the data-set reflections into 20 resolution bins and calculated the correlations per resolution bin. Finally, an average of the bin correlations up to ‘anomalous resolution’ was calculated. The anomalous resolution was determined once for each data set, corresponding to the lowest resolution (the largest number) included in those resolution bins in which the correlation between the multivariate *E* values and the final *E* values was smaller than 0.05 and an average of correlations from three consecutive resolution bins was smaller than 0.05.

Estimation of *E* values from Friedel pair differences (Δ*E*) was also implemented in *Afro* and was tested on the 182 SAD data sets to compare its performance against the multivariate estimation. Complete structure solution from Δ*E* was attempted with *Crank*2 using the same pipeline and default options as used in the runs from multivariate *Afro*.

The anomalous substructure obtained by *PRASA* is considered to be ‘correctly determined’ if at least one third of the atoms in the final anomalous substructure had a matching atom (within 2 Å distance) in the substructure obtained after transformation by *SITCOM* (Dall’Antonia & Schneider, 2006 ▸). Similarly to as in Skubák (2018 ▸), we have observed that typically if approximately 1/3 of the substructure atoms have been correctly identified in substructure determination, the remaining significant anomalous scatterers can either be added by *Crank*2 from the anomalous maps or their absence does not affect the success of model building.

The model-building performance is judged by the fraction of the PDB-deposited model backbone that is ‘correctly built’. A residue is considered to be correctly built if its C^α^ position is at a distance of at most 2 Å from a deposited model C^α^ (‘C^α^-deposited’) position and a neighbouring C^α^ position is at a distance of at most 2 Å from a neighbour of the C^α^-deposited position (sequence identity or directionality is not checked). A custom script evaluating the model-building performance using these criteria was used.

For all data sets where one of the pipelines failed to determine the substructure, a ‘thorough’ substructure-determination protocol was tested: the number of *PRASA* trials was increased to 100 000 trials from the default maximum of 2000 trials, more high-resolution cutoffs were tested (the high-resolution cutoff step was decreased to 0.1 from the default of 0.25) and the initial high-resolution cutoff was set to be identical to the anomalous resolution. The thorough protocol aims to estimate whether it is possible to determine the substructure by *PRASA* from the input *E* values at all.

## 3. Results and discussion

**Figure 1:**
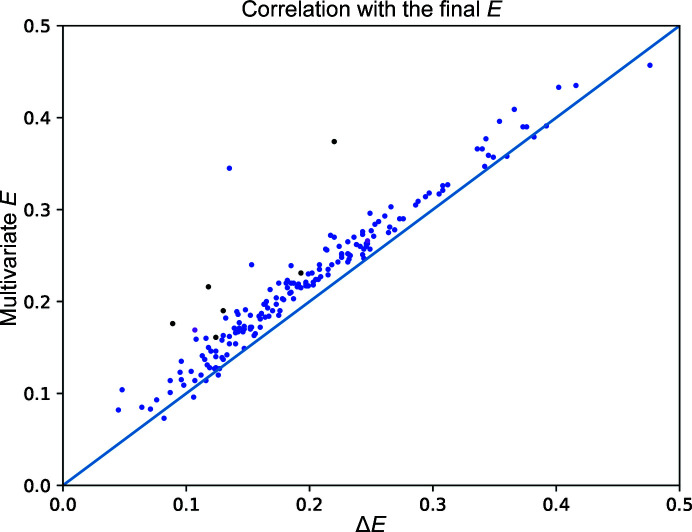
The correlation of Δ*E* (*x* axis) and multivariate *E* from *Afro* (*y* axis) with the ‘final’ substructure *E* for each of the 182 tested data sets. The data sets for which the substructure was correctly determined from the multivariate *E* but not from Δ*E* are displayed in black (comparing the results of the default substructure-determination protocol) and magenta (the ‘thorough’ substructure-determination protocol).

The correlation of the multivariate *E* values estimated by *Afro* with the final substructure *E* is typically significantly larger than that for Δ*E*, as demonstrated by Fig. 1 ▸. In tests on the 182 SAD data sets, the average correlation improved by 13% (from 0.197 to 0.223) and an improved correlation was observed for 94% of the data sets.

The overall better quality of the *E* estimates calculated by *Afro* allowed successful substructure determination by *PRASA* for six data sets that did not work using Δ*E*. As summarized in Table 1 ▸, the total number of data sets with the substructure correctly determined increased from 162 (89.0%) using Δ*E* to 168 (92.3%) using multivariate *Afro*. If these six data sets were removed from the comparison, the average fraction of the substructure that was correctly determined remained similar (0.774 versus 0.760). This indicates that the improvement in the quality of the multivariate *E* values from *Afro* may not be of great practical importance if the substructure can be obtained using the Δ*E* values; however, it may allow successful substructure determination for data sets where the substructure could not be determined using the Δ*E* values.

A majority of the model was correctly built for 156 data sets (85.7%) starting from substructure determination using Δ*E* and for 161 data sets (88.5%) starting from substructures determined by multivariate *E* from *Afro*.

Using the ‘thorough’ substructure-determination protocol with a large number of substructure trials and resolution cutoffs for the data sets where substructure determination failed led to the determination of another two substructures starting from the multivariate *Afro*. Similarly, one more substructure could be determined using the thorough protocol starting from Δ*E*; this substructure was obtained starting from the multivariate *E* using the default protocol.

In total (default + thorough protocol), seven substructures were determined from the multivariate *E* values that were not determined from the Δ*E* values. Furthermore, determination of one other substructure required the thorough protocol starting from Δ*E*, while the default protocol was sufficient if multivariate *Afro* was used. Analysis of the success rates for this data set (PDB entry 2pgc) shows that this was not a coincidence: only four solutions were obtained in 100 000 trials from Δ*E* (a success rate of 1 in 25 000) and 27 solutions were obtained using the multivariate *Afro* (1 in 3704).

The data sets used in this paper may not be fully representative of user data. In particular, a large fraction (almost 45%) of the data sets come from the automated JCSG pipeline (Elsliger *et al.*, 2010 ▸), which may differ from more recent data-collection methods. Furthermore, a limited number of data sets for which the structure could not be solved are included in the sample used for the paper; such data sets are typically neither deposited nor shared. Thus, the differences in results between the pipelines should not be considered as a quantitative estimate of success-rate improvement for user data but rather as qualitative evidence that the improved |*F*_a_| and *E* estimates by *Afro* may lead to successful substructure determination and model building for data sets where it failed using Δ*E*.

The multivariate |*F*_a_| estimation by* Afro* has been integrated into the *Crank*2 pipeline for automated structure solution from experimental phases and is distributed as part of the *CCP*4 package, which is available as a binary and as open source. 

## Figures and Tables

**Table 1:** Number of data sets for which the substructure was determined and the majority of the model was built by the two tested pipelines: starting from *E* calculated as Friedel pair differences and by multivariate *Afro* The first number in each cell denotes the number of successes using the default substructure-determination protocol and the second number that using the ‘thorough’ substructure-determination protocol with a substantially larger number of trials and a larger number of high-resolution cutoffs.

	No. of data sets (default/thorough)	
	Delta	Multivariate
Substructures determined	162/163	168/170
Models built	156/157	161/162

## Appendix A Derivation of the expected value of |*F*_a_|

The expected value of |*F*_a_| is calculated, by definition, from the conditional probability distribution *P*(|*F*_a_|; |*F* ^+^|, |*F* ^−^|),

The top and bottom integrals are the first and zeroth moments of the distribution *P*(|*F*_a_|; |*F* ^+^|, |*F* ^−^|), which can be obtained from the joint distribution of structure factors *F*_a_, *F* ^+^, (*F* ^−^)*, which can be approximated by a complex multivariate normal of mean zero and covariance Σ,

where *a*_*jk*_ and *b*_*jk*_ denote the real and imaginary components of the inverse covariance matrix. The zeroth, first and second moments of |*F*_a_| can be obtained by integrating out the unknown phase angles (α_a_, α^+^ and α^−^) and averaging over |*F*_a_|:

Changing variables (β = α^+^ − α^−^, φ = α^−^ − α_a_) leaves only an expression in β and φ; thus, α_a_ can be integrated out:

Using the formula  =  , the following equation results:

where

The integral over |*F*_a_| has an analytical solution:

To derive the equation that assumes that the phases of the Friedel pairs are equal while considering measurement errors in the observed structure-factor amplitudes, we assume that β = 0 and the equations reduce to the following:

Substituting equation (11) for *n* = 1 in the numerator of equation (3) and for *n* = 0 in its denominator and using the formulas  and Φ(1, 1, *x*) = exp(*x*), we obtain

In the case of the marginal distribution *P*(*F* ^+^, *F* ^−^) without the Friedel pair phase equality assumption, substituting *n* = 0 and using the relation Φ(1, 1, *x*) = exp(*x*) gives

The covariance matrix Σ is calculated as follows:

where  and  denote the measurement errors of |*F* ^+^| and |*F* ^−^|, respectively, Σ_*N*_ = (〈|*F* ^+^|^2^ + |*F* ^−^|^2^〉)/2, Σ_*H*_ = Σ(*f*_o_ + *f*′)^2^ + *f*′′^2^, *f*_o_ is the non-anomalous scattering factor of the anomalously scattering atom type, *f*′ and *f*′′ are the real and imaginary anomalous scattering factors, *k* is a refinable local scale factor and ɛ is a symmetry-related statistical weight of reflection counting how many times the symmetry operations map the reflection to itself. All of the covariance matrix terms and summations are calculated per resolution bin, except for  ,  and ɛ which are applied per reflection.

## Appendix B Complete list of PDB codes of the data sets used for testing

A total of 182 SAD data sets for 170 macromolecular structures were used for testing. The sample consisted of 169 data sets for 157 structures used by Skubák (2018 ▸): PDB entries 1c8u, 1djl, 1dpx, 1dtx, 1dw9, 1e3m, 1e42, 1e6i, 1fj2, 1fse, 1ga1, 1hf8, 1h29, 1i4u, 1lvy, 1lz8, 1m32, 1mso, 1ocy, 1of3, 1rgg, 1rju, 1vjn, 1vjr, 1vjz, 1vk4, 1vkm, 1vlm, 1vqr, 1z82, 1zy9, 1zyb, 2a3n, 2a6b, 2ahy, 2aml, 2avn, 2b78, 2b79, 2b8m, 2etd, 2etj, 2ets, 2etv, 2evr, 2f4p, 2fdn, 2fea, 2ffj, 2fg0, 2fg9, 2fna, 2fqp, 2fur, 2fzt, 2g42, 2g4h, 2g4j, 2g4k, 2g4l, 2g4m, 2g4n, 2g4o, 2g4p, 2g4q, 2g4r, 2g4s, 2g4t, 2g4u, 2g4v, 2g4w, 2g4x, 2g4y, 2g4z, 2g51, 2g52, 2g55, 2gc9, 2hba, 2ill, 2nlv, 2nuj, 2nwv, 2o08, 2o0h, 2o1q, 2o2x, 2o2z, 2o3l, 2o62, 2o7t, 2o8q, 2obp, 2oc5, 2od5, 2od6, 2oh3, 2okc, 2okf, 2ooj, 2opk, 2osd, 2otm, 2ozg, 2ozj, 2p10, 2p4o, 2p7h, 2p7i, 2p97, 2pg3, 2pg4, 2pgc, 2pim, 2pn1, 2ppv, 2pr7, 2prr, 2prv, 2prx, 2pv4, 2pw4, 2q2l, 2rkk, 2v0o, 3bpj, 3fki, 3gyv, 3k9g, 3km3, 3lmt, 3lmu, 3men, 3njb, 3o2e, 3oib, 3p96, 3s6l, 4us7, 4xvz, 4xxt, 4yf1, 5b82, 5gwd, 5ifg, 5irr, 5j4r, 5kjh, 5lg6, 5llw, 5loi, 5lsq, 5sus and 5suu, and three undeposited data sets. Furthermore, 13 more recent data sets for 13 different structures deposited in the previous few years were randomly chosen from the PDB and added to the sample: PDB entries 6kvr, 6tke, 6xjn, 6xqi, 6ygu, 6yrl, 7cdw, 7eiv, 7fad, 7fi4, 7lt1, 7oc3 and 7yx8.
